# Serum neurofilament light and tau as prognostic markers for all-cause mortality in the elderly general population—an analysis from the MEMO study

**DOI:** 10.1186/s12916-021-01915-8

**Published:** 2021-02-15

**Authors:** Nicole Rübsamen, Aleksandra Maceski, David Leppert, Pascal Benkert, Jens Kuhle, Heinz Wiendl, Annette Peters, André Karch, Klaus Berger

**Affiliations:** 1 Institute of Epidemiology and Social Medicine, University of Münster, Domagkstraße 3, 48149 Münster, Germany; 2 Neurologic Clinic and Policlinic, Departments of Medicine, Biomedicine and Clinical Research, University Hospital Basel, University of Basel, Petersgraben 4, 4031 Basel, Switzerland; 3 Clinical Trial Unit, Department of Clinical Research, University Hospital Basel, University of Basel, Spitalstr 12, 4031 Basel, Switzerland; 4 Department of Neurology with Institute of Translational Neurology, University Hospital Münster, Albert-Schweitzer-Straße 33, 48149 Münster, Germany; 5 Institute of Epidemiology II, Helmholtz Zentrum München, Ingolstädter Landstr 1, 85764 Neuherberg, Germany

**Keywords:** Biomarker, Mortality, Neurofilament, Prognosis, SIMOA, Tau

## Abstract

**Background:**

Neurofilament light chain (NfL) is a cytoskeletal protein component whose release into blood is indicative of neuronal damage. Tau is a microtubule-associated protein in neurons and strongly associated with overall brain degeneration. NfL and tau levels are associated with mortality in different neurological diseases, but studies in the general population are missing. We investigated whether NfL and tau serum levels could serve as prognostic markers for overall mortality in elderly individuals without pre-defined neurological conditions. Further, we investigated the cross-sectional associations between NfL, tau, neuropsychological functioning, and brain structures.

**Methods:**

In 1997, 385 inhabitants of Augsburg who were aged 65 years and older were included in the Memory and Morbidity in Augsburg Elderly (MEMO) study. They participated in a face-to-face medical interview including neuropsychological tests and magnetic resonance imaging (MRI) of the brain. NfL and tau were measured from non-fasting blood samples using highly sensitive single molecule array assays. To assess the prognostic accuracy of the biomarkers, concordance statistics based on the predicted 5-year survival probabilities were calculated for different Cox regression models. Associations between the biomarkers and the neuropsychological test scores or brain structures were investigated using linear or logistic regression.

**Results:**

NfL (HR 1.27, 95% CI [1.14–1.42]) and tau (1.20 [1.07–1.35]) serum levels were independently associated with all-cause mortality. NfL, but not tau, increased the prognostic accuracy when added to a model containing sociodemographic characteristics (concordance statistic 0.684 [0.612–0.755] vs. 0.663 [0.593–0.733]), but not when added to a model containing sociodemographic characteristics and brain atrophy or neuropsychological test scores. NfL serum levels were cross-sectionally associated with neuropsychological test scores and brain structures.

**Conclusions:**

The association between NfL serum levels and brain atrophy and neuropsychological performance in individuals without overt neurological disease is similar to that seen in patients with neurodegenerative diseases. These findings support the concept of a continuum of physiological aging and incipient, subclinical pathology, and manifest disease.

NfL, but not tau, serum levels might serve as a prognostic marker for all-cause mortality if no other clinical information is available.

**Supplementary Information:**

The online version contains supplementary material available at 10.1186/s12916-021-01915-8.

## Background

Neurofilaments (Nf) are cytoskeletal protein components whose release into cerebrospinal fluid (CSF) and blood is indicative of neuronal damage [1]. Neurofilament light chain (NfL) CSF levels are increased in several inflammatory and primarily degenerative neurological diseases that result in decreased function of speed and neuronal loss. Moreover, NfL CSF levels are associated with mortality of patients with parkinsonism [2], mitochondrial disease [3], and other neurodegenerative diseases [4, 5]. Similarly, this is the case for higher NfL serum levels in patients with sporadic Creutzfeldt-Jakob disease [6], after ischaemic stroke [7] and after spontaneous intracerebral haemorrhage [8].

Tau, a microtubule-associated protein in neurons, is complementary to NfL [9] because of its strong association with the process of overall brain degeneration. Tau has also been reported to be associated with mortality in patients with parkinsonism [2], sporadic Creutzfeldt-Jakob disease [6], and intracerebral haemorrhage [10] in recent years.

The development of single molecule array (SIMOA) assays [11] with increased sensitivity has allowed to define physiological levels of NfL and tau [12] so that subtle increases in the course of subclinical disease can be quantitated. Based on a strong correlation of biomarker levels in CSF and blood, this new technology allows to avoid lumbar puncture and enables high-throughput analysis of biomarkers in large population-based studies. In the Rotterdam study, plasma NfL was measured among 4000 non-demented adults above the age of 55 years [13]. A higher plasma NfL level was associated with a higher risk of all-cause dementia or Alzheimer’s disease. Mielke et al. report associations between plasma NfL and cognitive decline among 79 adults above the age of 50 years [14]. These two studies focus mainly on neuroimaging and cognitive outcomes, but not on all-cause mortality.

We investigated whether serum NfL and tau could serve as prognostic markers (alone or in combination with other factors) to predict absolute risks of overall mortality in elderly individuals without pre-defined neurological condition. Further, we investigated the cross-sectional associations between NfL, tau, neuropsychological functioning, and brain structures.

## Methods

### Selection and description of participants

The Memory and Morbidity in Augsburg Elderly (MEMO) is a 1997/1998 re-examination of participants from the 1989/1990 World Health Organization (WHO) Monitoring Trends and Determinants in Cardiovascular Disease (MONICA) Survey Augsburg, Germany [15, 16]. Initially in 1989, a random sample of the population of Augsburg, a city in southern Germany, was drawn from the office of registration. The sample’s age range was 25–74 years and the response for the MONICA survey was 76.8% (4940 participants). For the MEMO study 8 years later, we contacted all participants of the MONICA survey aged 65 years and older on July 1, 1997, and living in the city of Augsburg or two large, adjacent suburbs. The overall response proportion for the MEMO study was 60.6% (385 participants). Written informed consent was obtained from all MEMO participants. The joint ethics committee of the University of Muenster and the Westphalian Chamber of Physicians approved the study.

All participants received a face-to-face medical interview on prior and current diseases and risk factors with special emphasis on the history of neurological and psychiatric diseases. The diagnostic workup also included a standardized neurologic examination and the CASCADE neuropsychological test battery [17]. The examination was done by one of two study physicians specifically trained for 3 months in an outpatient clinic for movement disorders. It included parts of the Unified Parkinson’s Disease Rating Scale (UPDRS). The neuropsychological test battery was based on standardized and valid tests assessing the domains of short-term memory, cognitive speed, attention, and motor speed: Measures of short-term memory were derived from three word recall tests [18] and summarized in a single ‘memory score’. Cognitive speed and attention were assessed via the Stroop test [19] and the Letter Digit Substitution test [20]; they were summarized in a single ‘speed score’. Motor speed was assessed via the Purdue Pegboard test [21] (referred to as ‘motor score’ in the following). The three domain scores were summarized in a global cognitive function score, the CASCADE score [17].

The diagnoses of diabetes mellitus (type I or II), hypertension, and cardiovascular disease (stroke, transitory ischaemic attack (TIA), myocardial infarction, heart failure, arrhythmia) were self-reported during the interview and validated by medical records. Blood pressure and body mass were measured according to the MONICA protocol and intake of medication during the last 7 days was documented.

### Brain magnetic resonance imaging (MRI)

MRI scanning was performed during the study visit at baseline on a 1.5-T MR imager (Gyroscan NT, Philips, Leiden, The Netherlands) at the department of Radiology and Neuroradiology, Central Hospital of Augsburg, on average 1.5 h after drawing blood samples. Of the whole sample (*N* = 385), 117 individuals did not receive an MRI due to contraindications (artificial eye lenses or incorporated metal artefacts including pacemakers (*n* = 69), claustrophobia (*n* = 16), dissent (*n* = 11), or other reasons (*n* = 21)). The MEMO participants with MRI were on average younger (mean age 72.3 vs. 73.7) than those without MRI. The MRI protocol included proton density (PD)-, T1-, and T2-weighted images acquired with spin echo sequences with 20 axial slices, 5 or 6 mm thick with an interslice gap of 1 or 1.2 mm, respectively.

MRI reading for the MEMO study was done at the CASCADE radiology centre (Department of Radiology, Daniel Den Hoed Cancer Centre, Rotterdam, The Netherlands) by a single reader on hard copies using an established and reliable rating scale [22]. White matter lesions were considered present if these were hyperintense on both PD- and T2-weighted images and not hypointense on T1-weighted images. The rating scale assessed subcortical white matter lesions (WML) by number and three different sizes based on the diameter: ‘small’ if less than 3 mm, ‘medium’ if between 4 and 10 mm, and ‘large’ if greater than 10 mm. Lesions were counted per size and lobe. An approximate white matter lesion load was then calculated with the assumption of a fixed volume per category of lesion size (4.2 μL for small lesions, 113 μL for medium lesions, and 905 μL for a large lesion), enabling a quantitative measure for lesion load. Periventricular white matter lesions were rated semi-quantitatively per region: adjacent to the frontal horn (frontal capping), adjacent to the lateral wall of lateral ventricles (bands), and adjacent to the occipital horn (occipital capping) on a scale of 0 (no white matter lesions), 1 (pencil thin periventricular lining), 2 (smooth halo or thick lining), or 3 (large confluent white matter lesions). The overall degree of periventricular white matter lesions was calculated by adding up the scores for the three separate categories (range 0–9) [22, 23]. Lacunar infarction on MRI was defined as the presence of a hypointense (T1, PD) or hyperintense (T2) lesion of a diameter between 3 and 15 mm. Cortical atrophy was assessed on a semi-quantitative scale with 4 categories (0–3) rated for each lobe and the Sylvian fissure. These lobar ratings were subsequently summarized to a total cortical atrophy score with a range from 0 (no atrophy) to 15 (maximum atrophy) [22]. An atrophy score > 10 (representing the 75th percentile) was defined as the presence of severe cortical atrophy.

### Laboratory measurements

Non-fasting blood samples were collected from each consenting participant immediately after the interview. Samples were processed directly in the study centre within 2 h. After centrifugation, serum and plasma aliquots were prepared for long-term storage at − 80 °C. While the plasma aliquots had been depleted by other analyses in the previous decades, the serum aliquots had remained frozen for 22 years. Only serum aliquots were used in this analysis.

Serum NfL and tau were quantified with commercially available kits on the single molecule array HD-1 analyser (Quanterix, Lexington, MA, USA), as previously described [24, 25]. Samples were run in duplicate by board-certified technicians blinded to clinical information. The coefficients of variation for all samples reported were < 20%.

### Mortality assessment

Vital status of all MEMO participants and time of exit was documented prospectively until December 31, 2015, on which date the survival times of participants still alive were censored. The median follow-up time (quantified with the reverse Kaplan-Meier method [26]) was 17.9 years. Death certificates were obtained from local health departments and categorized in all-cause, cancer, cardiovascular, coronary heart disease, and stroke mortality. None of the participants was lost to follow-up.

### Statistical analysis

Age- and sex-dependency of NfL and tau serum levels were investigated using linear regression with a fractional polynomial for age [27]. In all other regression analyses, NfL and tau measurements were included as independent variables and were internally standardized according to the collected data, i.e. any effect estimate refers to an increase of one standard deviation (SD). Information on comorbidities (any histories of myocardial infarction, heart failure, vascular surgery, pacemaker, cancer, diabetes, joint replacement, or trauma with loss of function based on medical records) was taken into account in some analyses. Associations between any of the biomarkers and all-cause mortality were investigated using Cox regression models (adjusted for age, sex, years of education, and presence of any comorbidities). Subgroup analyses by sex were conducted according to Figueiras and colleagues [28]. Missing values in MRI measurements (covariates in the Cox models) were imputed (R package ‘mice’ [29] version 3.8.0) with 100 multiple imputations. Under the missing at random assumption, multiple imputation is typically more efficient than complete-case analysis (i.e. smaller standard errors), because it uses also information in incomplete cases. The imputation model included the event indicator and the Nelson-Aalen estimator of the cumulative hazard rate [30, 31], all variables that appear in the Cox regression models plus disease history (blood pressure, diabetes, surgeries of the brain, heart, or hip) and general health behaviour (smoking, alcohol consumption) as auxiliary variables. The fraction of incomplete cases among the observed, which is an estimate for the gain in precision when comparing multiple imputation to complete-case analysis [32], was 18% for each MRI variable. To assess the prognostic prediction performance of the biomarkers, concordance statistics (c statistic) [33] based on the predicted 5-year survival probabilities (estimated with 10-fold cross-validation) were calculated in each imputed dataset. The point estimate of the pooled c statistic was calculated as the average of the 100 individual estimates. The standard error was computed using both the within and between imputation variance of the c statistic. This way, the c statistic was calculated for different models: The initial model contained only terms for age at baseline, sex, and years of education. Eight further models were constructed by adding either a neuropsychological test score (memory, speed, motor, or CASCADE score), the atrophy score, the total volume of subcortical WML, or a biomarker to the initial model. The c statistic refers to the ability of a model to distinguish an individual with the endpoint (dead) from an individual without (alive). The c statistic indicates the probability that among two individuals, one dying within 5 years and one surviving, the individual bound to die will have a higher predicted risk than the surviving individual will [34]. Jack-knife estimation [35] was used to assess the c statistic improvement between nested models (R package ‘validstats’ [36] version 1.4). Whenever there was an improvement in the c statistic, the respective model was expanded by adding another predictor, e.g. by adding NfL to a model containing age, sex, years of education, and the CASCADE score. This stepwise approach was done because when statistical procedures are used to test for incremental prognostic information, the new biomarker should be tested for significance only after all other predictors have already been included in the model [37]. In the example above, the test of interest is whether NfL adds significantly to a model that already includes age, sex, years of education, and the CASCADE score, not whether the CASCADE score is chosen before NfL in a stepwise variable-selection process.

In a sensitivity analysis, the initial model contained comorbidities in addition to age, sex, and years of education. We also repeated these steps using stroke mortality as outcome to investigate the biomarkers’ prognostic value for death due to neurological disease. Stroke was the only neurological cause of death that had a reasonably high number of events. Only three deaths from dementia occurred in the MEMO study.

Associations between the biomarkers and the neuropsychological test scores were investigated using linear regression. Associations between the biomarkers and brain structures were investigated using logistic regression (binary outcomes were the presence of large WML and lacunar strokes), ordinal logistic regression (size of WML categorized as none, medium, or large), or linear regression (atrophy score). All regression analyses were adjusted for age, sex, years of education, and presence of any comorbidities. Missing values in MRI measurements (outcomes in these regression models) were not imputed because standard errors from multiple imputation are likely to be larger than those from complete-case analysis when only the outcome variable has missing values [38]. Additional to logistic regression, discrimination between participants with large WML versus those without, or those with lacunar strokes versus those without was measured by the area under the curve (AUC) corrected for over-optimism with 1000 bootstrap repetitions (R package ‘rms’ [39] version 5.1-4). The closer the AUC is to one, the better is the discrimination. Additional to linear regression, the effect size was calculated as a measure of how much variation in the outcome could be explained by the biomarker. The calculation of effect sizes was based on partial *η*^2^. Unlike adjusted *R*^2^, which measures the contribution of the entire model in explaining the variance, partial *η*^2^ measures the contribution of the individual independent variable. A partial *η*^2^ equal to or greater than 0.01 presents a small effect, equal to or greater than 0.06 presents a medium effect, and equal to or greater than 0.14 presents a strong effect [40]. All analyses were performed with R [41] version 3.6.1.

## Results

### Participants

Among all 385 participants, 47.3% were female (Table 1). The median age at baseline was 73 years (interquartile range 69–76 years). One or more comorbidities (as defined in the ‘Methods’ section) were present among 26.5% of the participants.

**Table 1:** Participant characteristics

	All (***N*** = 385)	MRI available (***N*** = 268)	No MRI (***N*** = 117)
Age (years)	73.0 (69.0, 76.0)	72.0 (69.0, 76.0)	74.0 (70.7, 77.0)
Women	182 (47.3)	128 (47.8)	54 (46.2)
Years of education	10.0 (10.0, 11.0)	10.0 (10.0, 11.0)	10.0 (10.0, 11.0)
Body mass index (kg/m^2^)	27.7 (25.2, 29.8)	27.8 (25.6, 29.8)	27.2 (24.8, 30.1)
Alcohol consumption (g/day)	5.8 (0, 22.9)	5.7 (0, 22.8)	6.0 (0, 23.8)
ADL score (scores 0–10)	0 (0, 1.0)	0 (0, 1.0)	0 (0, 2.0)
Systolic blood pressure (mmHg)	144.0 (133.0, 155.0)	143.0 (132.2, 154.0)	146.0 (135.0, 159.0)
History of arrhythmia	117 (34.4)	74 (31.2)	43 (41.7)
History of brain surgery	6 (1.6)	2 (0.8)	4 (3.5)
History of heart surgery	18 (4.7)	7 (2.6)	11 (9.6)
History of hip replacement	18 (4.7)	11 (4.1)	7 (6.1)
Any comorbidities*	102 (26.5)	61 (22.8)	41 (35.0)
Memory score (*z*-score)	− 0.1 (− 1.8, 2.0)	0.1 (− 1.6, 2.2)	− 0.7 (− 2.6, 1.4)
Speed score (*z*-score)	0.6 (− 1.8, 2.4)	0.6 (− 1.4, 2.5)	0.4 (− 2.4, 2.1)
Motor score (*z*-score)	0.2 (− 1.5, 1.8)	0.5 (− 1.4, 1.8)	− 0.2 (− 1.6, 1.4)
CASCADE score (*z*-score)	1.1 (− 3.6, 5.1)	1.7 (− 3.3, 5.6)	− 0.6 (− 4.8, 3.2)
Atrophy (scores 0–15)	9.0 (7.0, 10.0)	9.0 (7.0, 10.0)	–
Subcortical atrophy at the level of the anterior ventricular horns	0.3 (0.3, 0.4)	0.3 (0.3, 0.4)	–
Total volume WML (mL)	0.3 (0.1, 2.0)	0.3 (0.1, 2.0)	–
Any lacunar strokes	41 (15.3)	41 (15.3)	–
Serum NfL (pg/mL)	15.7 (12.1, 20.4)	15.5 (12.2, 19.9)	16.2 (11.7, 20.6)
Serum tau (pg/mL)	0.7 (0.5, 1.0)	0.7 (0.5, 1.0)	0.8 (0.5, 1.0)

Data are medians (25th percentile, 75th percentile) or numbers (percentage)

*Any histories of myocardial infarction, heart failure, vascular surgery, pacemaker, cancer, diabetes, joint replacement, or trauma with loss of function based on medical records

### Age- and sex-dependency

NfL serum levels were linearly dependent on age (*β* = 0.72 pg/mL per 1-year increase, 95% confidence interval 0.49–0.95 pg/mL, *p* < 0.001). There was no dependence on sex (*β* = 0.13 [− 1.92–2.19] pg/mL for men vs. women, *p* = 0.90). Tau serum levels were associated neither with age (*β* = 0.004 [− 0.006–0.01] pg/mL, *p* = 0.43) nor with sex (*β* = 0.03 [− 0.07–0.12] pg/mL, *p* = 0.59). NfL and tau levels were only slightly correlated (Pearson’s *ρ* = 0.12, *p* = 0.021).

### Mortality

**Fig. 1:**
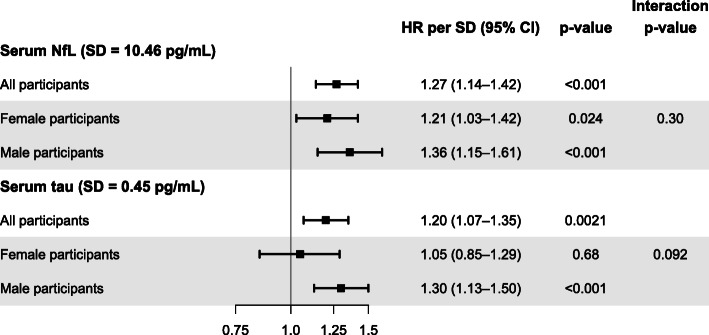
Results of Cox regression analyses for all-cause mortality

Both NfL and tau serum levels were associated independently with all-cause mortality: Per one SD increase, the risk of death increased by 27% and 20%, respectively (Fig. 1). A one SD increase reflected 10.5 pg/mL for NfL and 0.5 pg/mL for tau. Effect estimates were higher among men than among women. When focussing on stroke mortality only, serum NfL had a higher hazard ratio (1.58 [1.31–1.90]), but serum tau had a lower hazard ratio (1.02 [0.68–1.54]). In contrast, when focussing on non-stroke mortality only, both NfL (1.15 [1.01–1.31]) and tau serum levels (1.15 [1.03–1.28]) were still associated independently with all-cause mortality.

**Fig. 2:**
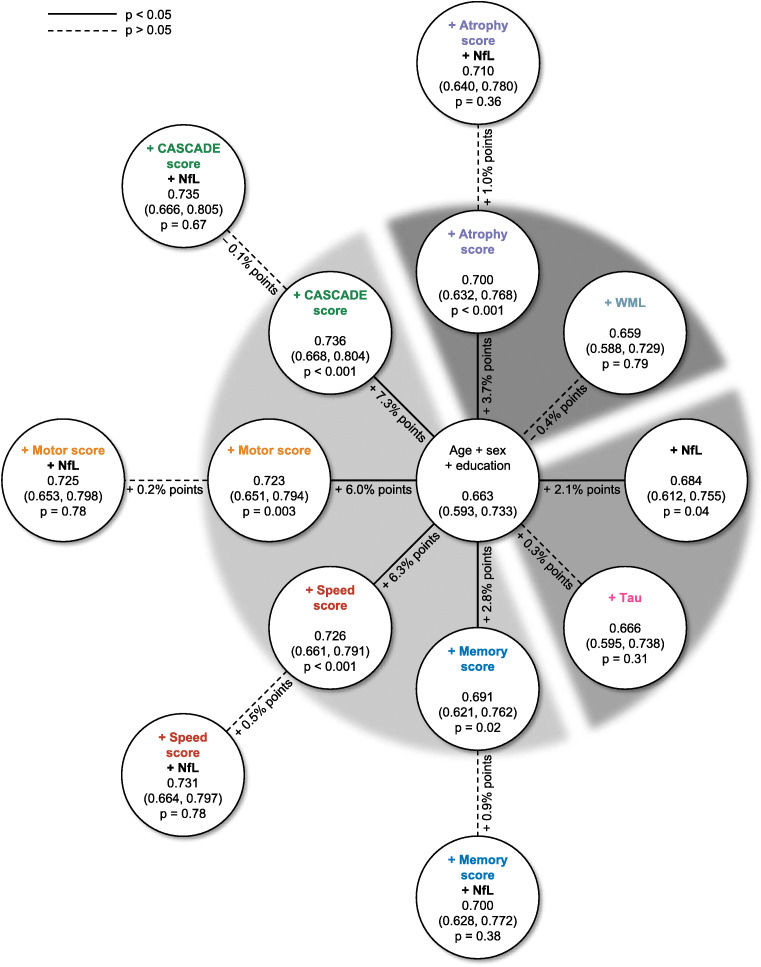
c statistic for all-cause death within 5 years after the study visit

When adding a biomarker to a model containing sociodemographic characteristics (age, sex, years of education) only, the prognostic accuracy for all-cause death within 5 years increased significantly for NfL (c statistic 0.663 [0.593–0.733] vs. 0.684 [0.612–0.755], *p* = 0.04), but not for tau (Fig. 2). Although there were interactions with sex on the relative risk scale, adding these interactions to the prognostic models did not improve the c statistics. NfL did not improve prognostic accuracy when added to a model containing sociodemographic characteristics and atrophy or neuropsychological test scores. Cognitive function measured by the CASCADE score was the single variable that increased prognostic accuracy the most. When adding a biomarker to a model containing comorbidities in addition to sociodemographic characteristics, the absolute increase in prognostic accuracy was similar to the models without comorbidities (Additional file 1), e.g. NfL increased the c statistic by 0.021 when added to a model containing sociodemographic characteristics only and by 0.020 when added to a model containing sociodemographic characteristics and comorbidities. When focussing on stroke mortality, the incremental increase of prognostic accuracy when adding NfL was smaller in relation to neuropsychological test scores (Additional File 2).

### Neuropsychological tests and brain structures

NfL serum levels were associated with the CASCADE score as well as with its domains cognitive and motor speed, but not with the domain memory (Table 2). Effect sizes were small for all associations. Tau levels were not associated with any neuropsychological test score.

**Table 2:** Results of linear regression analyses for neuropsychological tests and atrophy score

	***β*** per SD (95% CI)	***p*** value	Partial ***η***^**2**^
**Memory score**			
Serum NfL	− 0.27 (− 0.56–0.02)	0.067	0.0096
Serum tau	0.10 (− 0.17–0.37)	0.46	0.0016
**Speed score**			
Serum NfL	− 0.41 (− 0.74–0.08)	0.014	0.017
Serum tau	0.04 (−0.27–0.35)	0.8	0.00018
**Motor score**			
Serum NfL	− 0.46 (− 0.70–0.21)	< 0.001	0.036
Serum tau	0.01 (− 0.22–0.25)	0.92	0.000031
**CASCADE score**			
Serum NfL	− 1.15 (− 1.83–0.47)	< 0.001	0.031
Serum tau	0.14 (− 0.50–0.78)	0.66	0.00056
**Atrophy score**			
Serum NfL	0.34 (0.05–0.64)	0.023	0.02
Serum tau	0.17 (− 0.13–0.47)	0.27	0.0048
**Subcortical atrophy at the level of the anterior ventricular horns**			
Serum NfL	0.001 (− 0.004–0.006)	0.64	0.00084
Serum tau	0.005 (0.00002–0.009)	0.049	0.015

All analyses were adjusted for age, sex, education, and any comorbidities. Partial *η*^2^ ≥ 0.01: small effect size. Partial *η*^2^ ≥ 0.06: medium effect size. Partial *η*^2^ ≥ 0.14: strong effect size

NfL serum levels were associated with overall brain atrophy score in the cross-sectional analysis (*β* = 0.34 [0.05–0.64] points increase of atrophy score per one SD increase of NfL), albeit with a small effect size (partial *η*^2^ = 0.02, Table 2). Tau was associated (small effect size) with subcortical atrophy at the level of the anterior ventricular horns (Table 2), but not with other brain structures (Table 3). The association between NfL and white matter lesions was more pronounced, with a 72% increased risk of a large lesion per one SD increase (Table 3). A similar result was observed for lacunar strokes with a 67% increased risk per one SD increase of NfL; in contrast, there was no association between WML and tau. Discrimination between participants with versus without large WML or lacunar strokes, as measured by the AUC of the models including NfL, sociodemographic characteristics, and any comorbidities, was moderate (0.66–0.71).

**Table 3:** Results of (ordinal) logistic regression for white matter lesions and lacunar strokes

	OR per SD (95% CI)	***p*** value	AUC
**WML (ordinal)**			
Serum NfL	1.44 (1.08–2.02)	0.022	
Serum tau	1.01 (0.80–1.28)	0.93	
**Large WML (binary)**			
Serum NfL	1.72 (1.24–2.51)	0.0024	0.71
Serum tau	0.99 (0.75–1.30)	0.95	0.68
**Any lacunar strokes (binary)**			
Serum NfL	1.67 (1.25–2.34)	0.0014	0.67
Serum tau	1.05 (0.73–1.46)	0.77	0.57

All analyses were adjusted for age, sex, education, and any morbidities

## Discussion

In this population-based study of elderly individuals without pre-defined neurological disease, we show that NfL, but not tau, serum levels can serve as a prognostic marker for all-cause mortality if no other clinical information is available. We also found cross-sectional associations between NfL and certain domains of neuropsychological functioning as well as brain structures.

Our results show that in population-based settings, NfL and tau serum levels can be used to identify individuals at higher risk of mortality (reflected by the hazard ratios above one) that are clinically normal, relative to a reference group. Our data establish a continuum of predictive value for NfL between subclinical morbidity and individuals with neurological disease [6–8]. Moreover, none of the patient- or population-based studies so far investigated the influence of NfL on absolute risks. Here we demonstrate that adding NfL to a model based on sociodemographic characteristics increased its discriminative ability by two percentage points: The probability that among two individuals, one dying within 5 years and one surviving, the individual bound to die will have a higher predicted absolute risk than the surviving individual is 66.3% for the model without and 68.3% for the model with NfL. The latter model yielded a similar c statistic to the model containing sociodemographic characteristics and the memory score. If a model already included neuropsychological test scores or MRI measurements, NfL did not add to the prognostic accuracy for mortality.

If, however, MRI measurements or a full (sub-)clinical morbidity profile are not available, serum NfL can be used instead for prognostic models and seems to harbour important health information in the general population. This is especially important since serial MRI measurements are often not available because of contraindications or time constraints, are costly, and do not cover the entire central nervous system.

When focussing on stroke mortality, the incremental increase of prognostic accuracy when adding NfL was smaller in relation to neuropsychological test scores. This was to be expected because NfL is an indicator of any axonal damage and is, therefore, not disease-specific. Although neuropsychological tests are easy to administer, they are also time-consuming and not applied as screening procedures to the general population. If confirmed and expanded, serum NfL may be the prognostic marker of choice if blood samples are taken within the routine check-up of elderly individuals, i.e. serum NfL may serve as an alternative to MRI in the outpatient setting.

NfL serum levels were significantly associated with brain structures involving small vessel vascular disease, i.e. large white matter lesions and lacunar strokes, at the time of assessment, but serum tau was not. This is in line with findings from a study that used NfL and tau measured in the CSF [42]. NfL was also associated with global brain atrophy while only tau had a small effect size on subcortical atrophy measures. Serum NfL has already been reported as a predictor of brain atrophy in multiple sclerosis [43]. We confirm these findings for healthy individuals.

### Strengths and limitations

The long follow-up for vital status with a considerable high proportion diseased among the study participants is a strength of our study. Based on the population-based sampling scheme and the high response rate at baseline, the MEMO study population is considered highly representative for the general German population in the respective age group. The MEMO study was also among the first ones that used MRI on a population level; therefore, we were able to include MRI results into the analysis.

Among the limitations is the small sample size that reduced the statistical power for several analyses. In particular, it was not possible to investigate the prognostic value of the biomarkers for predicting death from neurological disease because only three deaths from dementia occurred in the MEMO study. Increased NfL serum levels have also been reported in patients with acquired peripheral neuropathies [44], which are rarely assessed in population-based cohort studies because of the thorough workup needed, including nerve conduction studies [45]. We were therefore not able to exclude actively these individuals in our analyses. However, diabetes is the leading cause of peripheral neuropathy [45]. We adjusted all regression analyses for ‘any comorbidities’, which includes diabetes (i.e. among all individuals with comorbidities, 40% reported a physician’s diagnosis of diabetes). We thereby accounted for the majority of undiagnosed peripheral neuropathy cases. We also compared c statistics of models with and without ‘any comorbidities’ (Fig. 2 and Additional File 1). The absolute increase in prognostic accuracy when adding NfL was similar in both models. MRI reading was performed on hard copies, which is no longer done in the digital era. However, at the time of MRI reading for the MEMO study, the reader followed an established and reliable protocol.

## Conclusions

We found that both NfL and tau serum levels were associated with all-cause mortality even when age, sex, and education were taken into account. However, NfL serum levels had a better prognostic value and a larger effect size compared to tau. If comorbidities that were assessed by a medical interview were additionally taken into account, the predictive power of NfL serum levels was attenuated. In situations where a medical history is not available or comorbidities are in subclinical stage, however, NfL serum levels may provide important information for healthcare interventions or the prediction of mortality.

## Supplementary Information

**Additional file 1.** c statistic for all-cause death within 5 years after study visit (sensitivity analysis).

**Additional file 2.** c statistic for stroke death within 5 years after study visit.

## Data Availability

The datasets generated and/or analysed during the current study are available from the corresponding author on reasonable request.

## Abbreviations

AUC: Area under the curve
CSF: Cerebrospinal fluid
MEMO: Memory and Morbidity in Augsburg Elderly
MONICA: Monitoring Trends and Determinants in Cardiovascular Disease
MRI: Magnetic resonance imaging
NfL: Neurofilament light chain
SD: Standard deviation
SIMOA: Single molecule array
TIA: Transitory ischaemic attack
UPDRS: Unified Parkinson’s Disease Rating Scale
WML: White matter lesions
