# Supplementary figures and images for: Serum neurofilament light and tau as prognostic markers for all-cause mortality in the elderly general population—an analysis from the MEMO study
Source: BMC Med. 2021 Feb 15;19:38. doi: 10.1186/s12916-021-01915-8 (PMC7883435; doi:10.1186/s12916-021-01915-8)

—  $p < 0.05$   
- - -  $p > 0.05$

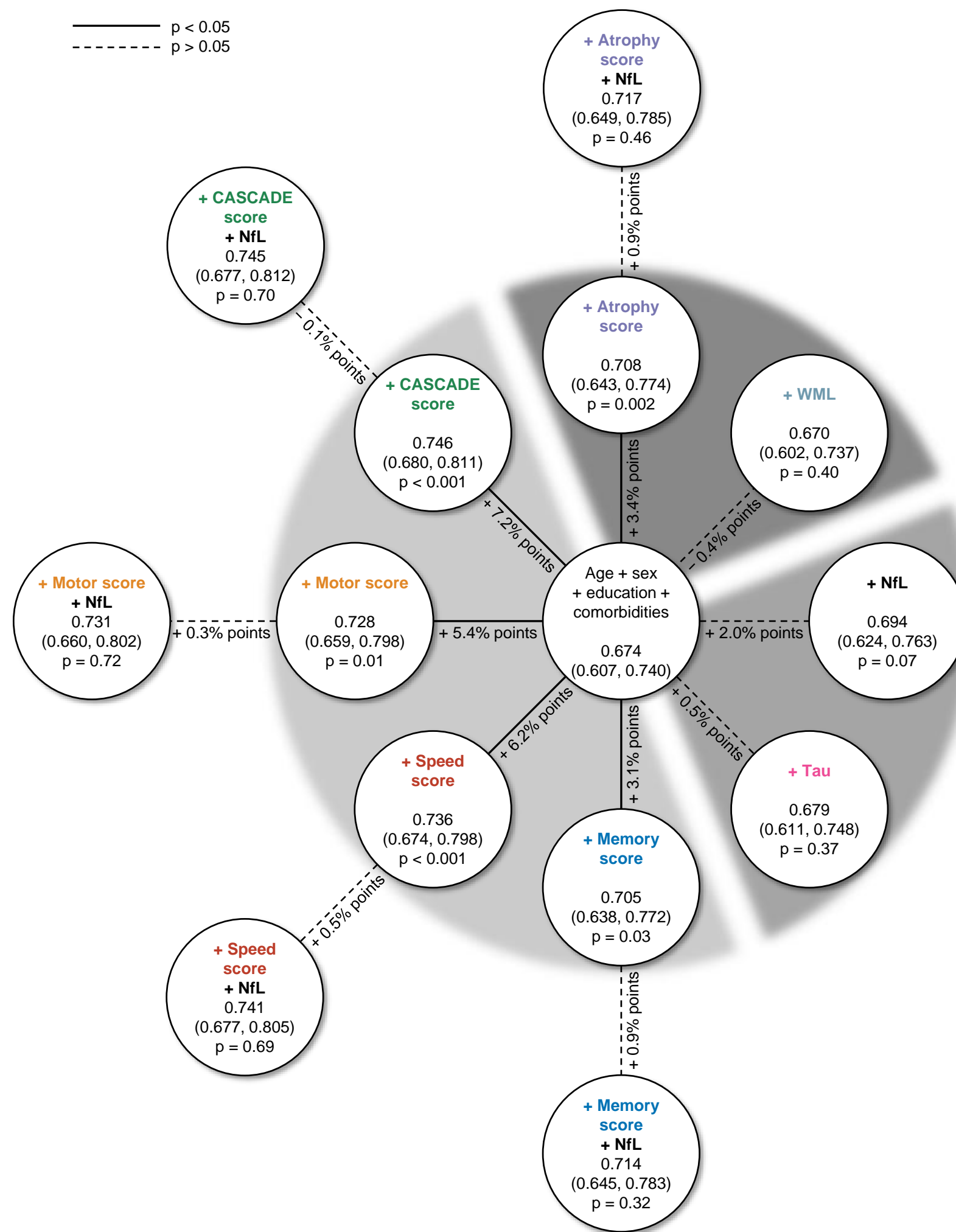

Supplement: Supplementary file 1 — Additional file 1. c statistic for all-cause death within 5 years after study visit (sensitivity analysis). [file 12916_2021_1915_MOESM1_ESM.pdf]

—  $p < 0.05$   
- - -  $p > 0.05$

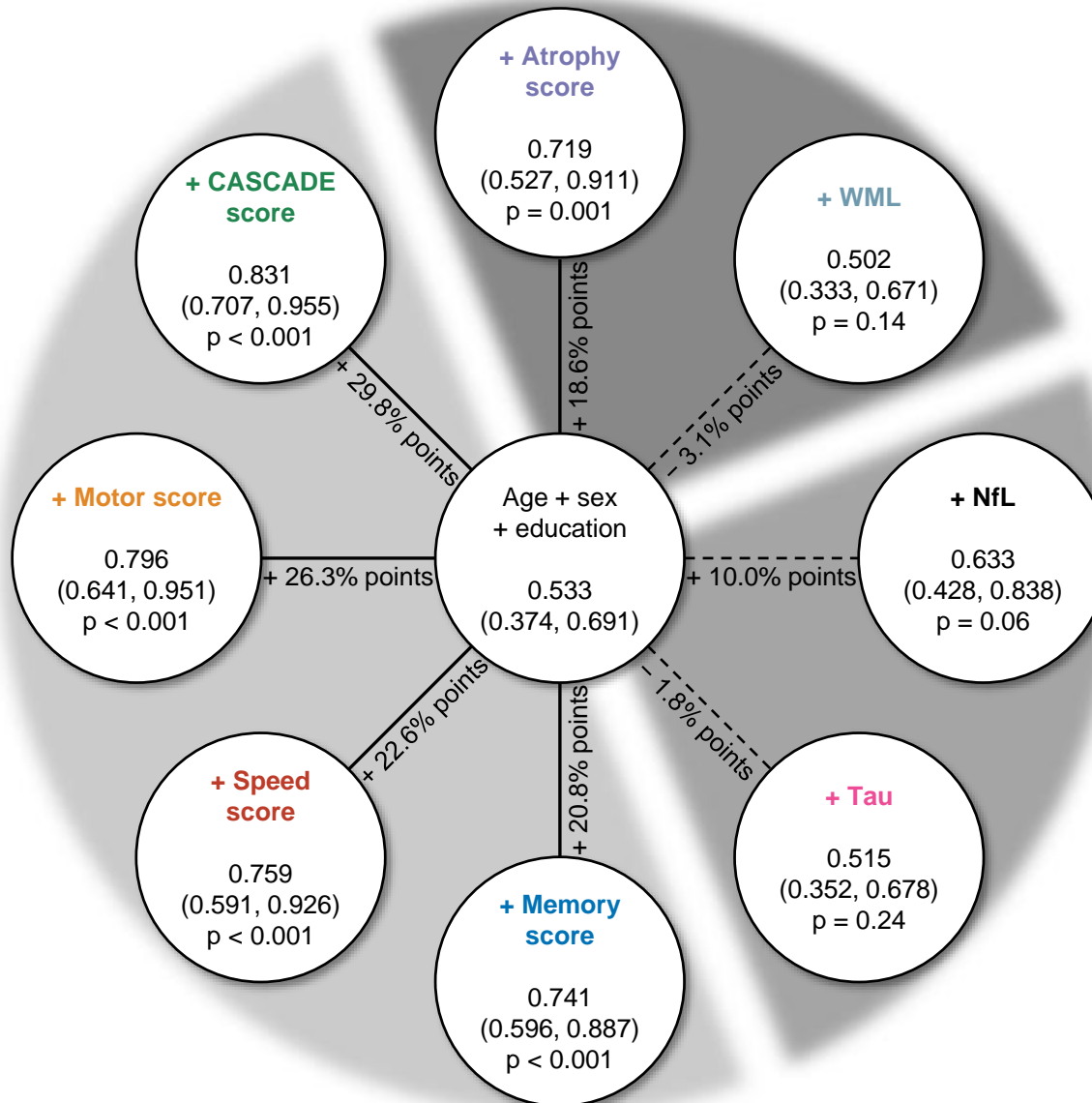

Supplement: Supplementary file 2 — Additional file 2. c statistic for stroke death within 5 years after study visit. [file 12916_2021_1915_MOESM2_ESM.pdf]
